# High lactic acid levels in the brain contribute to the generation of focal slowing on the electroencephalogram

**DOI:** 10.3389/fneur.2024.1393274

**Published:** 2024-04-17

**Authors:** Dmitry Alexandrovich Chegodaev, Polina Alekseevna Pavlova

**Affiliations:** ^1^Laboratory for Brain and Neurocognitive Development, Ural Institute of Humanities, Ural Federal University named after the First President of Russia B. N. Yeltsin, Yekaterinburg, Russia; ^2^Science Center for Cognitive Research, Sirius University of Science and Technology, Sirius, Russia

**Keywords:** lactate, acidosis, focal slowing, electroencephalogram, structural lesions, epilepsy

## Abstract

Focal slowing on the EEG is often associated with structural pathology of the brain. Despite the clinical significance of focal slowing, the actual electrochemical mechanisms underlying this EEG phenomenon are still poorly understood. This paper briefly reviews the role of lactate in the pathogenesis of brain disorders that are primarily related to focal EEG slowing. An attempt is made to trace the hypothetical link between this EEG pattern and focal cerebral tissue lactacidosis.

## Introduction

Focal slowing (0.5–7.9 Hz) is the electroencephalographic (EEG) pattern associated with focal structural or, to a lesser extent, with functional brain abnormalities. There is no specific etiology for this pattern. The most frequent etiological factors include: cerebral ischemia, cortical malformation, nonspecific gliosis (including reactive gliosis after stroke, and traumatic brain injury), tumors, hippocampal sclerosis, migraine, hypoglicemia, postictal state, and cortical malformations (1, 2). Focal slowing is one of the most common clinically significant EEG abnormalities. In particular, focal EEG slowing could roughly reflect the tumor aggressiveness (malignancy). For instance, glioblastoma, or metastatic brain tumors, are associated with focal slowing occurring in the delta (1–4 Hz) frequency band. In contrast, benign and slow-growing neoplasms such as meningiomas typically exhibit focal slowing in the theta (4–7 Hz) band (1). In spite of the obvious significance of this pattern, to the best of our knowledge, no studies or reviews have been conducted and published on the electrochemical aspects of focal EEG slowing.

The wide diversity of etiologies of this EEG pattern suggests that there should be a mechanism that is common across all brain disorders. One of the best candidates for this role is lactic acidosis, as it is a universal pathophysiological phenomenon.

This paper represents a set of biological evidence in favor of the speculative view that focal slowing in the EEG reflects local cerebral tissue lactic acidosis. The following discussion will concentrate on the most frequent pathological conditions associated with focal EEG slowing.

## Lactic acidosis in epileptic brain

Focal and diffuse slowing is the most typical and common postictal visual EEG abnormalities. EEG slowing is usually observed immediately after seizures and/or epileptiform discharges. Meanwhile, the most prominent feature of postictal brain metabolism is the elevated brain lactate. Strictly speaking, increased slow-wave activity as well as focal accumulation of lactate can be registered during both ictal and especially postictal periods (3), as confirmed by the use of intracranial EEG. Lactate accumulation in the epileptic brain exhibits a clear and consistent association with extracellular glutamate, which significantly elevates during the ictal and interictal periods (4). At least some of this glutamate is captured by astrocytes via transporters (GLT-1); this in turn triggers glycolysis and increased production of lactate (5).

In this regard, it is important to mention that lactate tissue acidosis is widely recognized as a factor in terminating seizures and epileptic activity. Thus, it can be assumed that lactate begins to accumulate during the seizure, and subsequent elevation of the lactate level contributes to the seizure termination.

Increased levels of lactate together with ammonia and creatine kinase are also revealed in the serum after seizures (6). And this has provided some grounds to suggest that an increased lactate level is a consequence of excessive muscle activity during a seizure. But a strong counterargument is a demonstration of increased brain lactate in paralyzed animals (3). Furthermore, one can assume that increased ammonia is lactacidosis-promoting factor, since fast ammonia accumulation in neural tissue may induce glycolysis and thus additional excessive lactate formation.

## Lactic acidosis in structural brain abnormalities

Among the major categories of diseases characterized by focal structural changes in the brain are cerebral ischemia (including stroke), cortical malformations, and disorders typically associated with gliosis.

During focal cerebral ischemia, brain lactate concentration increases. And this is explained by the fact that ischemia activates anaerobic glycolysis, the end product of which is lactic acid. Lactic acid [CH3CH(OH)COOH] at physiological pH, is almost completely dissociated to lactate [CH3CH(OH)COO^−^] and H^+^. Accumulation of lactate causes alterations in the dissociation of water and weak acids and leads to metabolic (lactic) acidosis (7). Apparently, the elevation of tissue lactate concentration depends on the severity and time from the onset of the brain hypoxia and energy depletion (8). It is known that the early effect of hypoxia is an initial hyperpolarization, which is provided by potassium (K^+^) channels: ATP-sensitive channels (KATP) and, to a less extent, calcium-activated K^+^ channels. Activation of KATP channels in the brain during hypoxia and ischemia by lactate and as a result of an alteration of the submembrane ATP/ADP ratio leads to an increase in K^+^ efflux. At an acidic pH, an additional factor influencing KATP could be the protonation of the C-terminal histidine residue (H216), which affects the polyamine-binding site. These conformational changes of KATP-channels prevent their polyamine block, promoting thus K^+^ efflux (9). On the one hand, hypoxic lactacidosic can lead to the activation of Na^+^/H^+^ exchanger 1 (NHE1) due to a reduction in intracellular pH, which results in sodium (Na^+^) influx (10). On another hand, there is evidence to suggest that the activity of NHE1 in excitable tissues highly depends on the metabolic status of the cells. It has been demonstrated that even mild hypoxia inhibits NHE1 activity (11). Moreover, after an initial period of metabolic inhibition, NHE1 can undergo post-translational modifications, which provide long-lasting reduction in NHE1 activity that has persisted after re-oxygenation (11). Thus, acute severe or prolonged depletion of cellular ATP can restrict Na^+^ influx for a long period. Additionally, ATP depletion due to oxygen glucose deprivation as well as local tissue acidosis have been recognized to increase chloride (Cl^−^) concentration in neurons (12, 13). Taking all mentioned above into account and bearing in mind the classical Goldman–Hodgkin–Katz voltage equation, it could be concluded that the value of neuronal membrane potential (Em) during lactacidosis becomes more negative, which corresponds to an increase in the hyperpolarization phase of the wave and the promotion of slow-wave activity as a whole. In the case of reduced volume of hypoxic tissue and related lactacidosic these processes manifest as focal slowing in the EEG.

Accumulation of lactic acid and extracellular lactacidosic is a hallmark of solid tumor microenvironment that promotes tumor growth, invasion, and metastasis. It is known that extracellular lactate in cancer directly correlates with tumor malignancy, as mentioned above. Increased aerobic glycolysis in tumor cells and augmented lactate production as a result, even in the presence of oxygen, is known as the Warburg effect. This effect is the main factor that supports tumor malignancy and aggressiveness (14). In the light of the above, it is tempting to conclude that the level of lactate accumulation and acidification in brain tumors influences the frequency of EEG focal slowing. One of the mechanisms by which the Warburg effect is realized is hydroxycarboxylic acid receptor 1 (HCA1R)-mediated signal transduction pathway. Lactate produced by cancer cells can act as a signal molecule, activating HCA1R. Receptor activation stimulates intracellular signaling pathways that facilitate tumor growth, immune evasion, and metastasis (15, 16). It is remarkable that HCA1R is tightly involved in the regulation of neuronal activity. It has been proven that activation of HCA1R in cortical neurons from human epileptic tissue causes a significant reduction in calcium spiking activity in these neurons (17). This finding is well complemented by the fact that a decrease in calcium spiking activity of pyramidal cortical cells correlates with EEG slow-wave activity (18). Downmodulation of neuronal activity by HCAR1 appears to be provided by the Gi-dependent intracellular adenylyl cyclase—cAMP—protein kinase signaling pathway. Moreover, HCAR1 interacts with GABAB receptors, which could supply additive neuronal inhibition (19). It is worth noting that this mechanism appears to be independent of the levels of ATP produced (20). This can serve as an argument that lactic acidosis, rather than hypoxia, is the primary factor determining the generation of regional EEG slowing. Finally, one study reported that intracellular lactate signaling promotes glutamine uptake and metabolism in cancer cells, leading to increased glutamate concentrations (21). This could also be one additional mechanism that results in hyperpolarization. One can speculate that in the case of neural tissue, glutamate released during excessive neuronal activity could act with non-NMDA receptors (possible Gluk2) on astrocytes, which triggers the release of ATP predominantly via connexin 43 hemichannels (Cx43) and activates a P2Y2/KATP cascade in neurons (22, 23).

The role of brain lactate in reactive gliosis (RG) is undisputable. It is widely recognized that increasing brain lactate concentration is considered a trigger factor for astrogliosis. However, it is not sufficiently clear how lactic acidosis is involved in the pathogenesis of chronic gliosis. In our view, this issue could be explained by the impairment of the astrocyte-neuronal lactate shuttle (ANLS). According to the ANLS hypothesis, glutamate, released from neurons, is taken up by astrocytes together with Na^+^. This in turn leads to the activation of Na^+^/K^+^-ATPase ATPase, which initiates glucose uptake in astrocytes. And the consequence is that glycolysis is stimulated in astrocytes, leading to an increase in lactate concentration. Lactate is released from astrocytes through the monocarboxylate transporter MCT4 into the extracellular space and is taken up by neurons through MCT2 and converted into pyruvate for ATP generation (24). It is known that RG is a prolonged process that includes neural tissue remodeling in peri-lesion perimeters. Tissue remodeling is known to involve several mechanisms, among them: (1) down-regulation of glutamine synthetase, which results in reducing the conversion of glutamate to glutamine in astrocytes; (2) increased expression of xCT (cystine/glutamate antiporter) (25). This may lead to an increase in extracellular glutamate and increase its utilization as a substrate in ANLS, which results in lactic acid overproduction.

Ischemic lesions and gliosis are the most typically associated pathoanatomic correlates of focal EEG slowing. Surprisingly, in the one study devoted to examining the structural substrates of focal EEG slowing, cortical malformations (CMs) occupied a leading place (2). Since CMs are common causes of epileptic discharges and seizures, one can assume that focal slowing has a postictal nature. A good illustration of this is the study research of patients with CM using ^1^H-magnetic resonance spectroscopy (^1^H-MRS) for detecting biochemical abnormalities. A study revealed enhanced lactate signal in patients who had seizures near the time of ^1^H-MRS examination (26). Occasionally, the enhanced lactate signal persists for a rather long time, that allows connecting it with the interictal EEG abnormalities. It has been speculated that the elevation of lactate in the brain tissue in CM could reflect epileptiform activity even in the absence of clinical seizures (27). However, the paradigmatic correlate of any kind of epileptiform discharges is a strong depolarization. Therefore, increased brain lactate and tissue acidosis cannot be a biochemical correlate of epileptiform activity. One of the key proposed mechanisms involved in reducing neuronal excitability requires the activation of interneurons by lactate through acid-sensing ion channel-1a (ASIC1a) (28). Importantly, ASIC1a-mediated pathway is realized only in pathological conditions since the lactate concentration required for ASIC1a activation must be supraphysiological (29). It should be stressed that the proton sensing sites of ASIC1a are located in the extracellular loop; therefore, channel activation first of all depends on extracellular acidification. In conditions of acidosis, protonation initiates structural rearrangements (fast conformational changes) in several extracellular sites of ASIC1a, leading to the opening of the channel gate (30). Opening of the ASIC1a gate potentiates a large Na^+^ and Ca^2+^ influx and depolarization of primarily GABAergic neurons (31), resulting in GABA release. Released GABA in turn activates γ-aminobutyric acid receptors. Neuronal inhibition is classically thought of as increasing the influx of Clˉ through γ-aminobutyric acid sub-type A receptors (GABAARs), leading to hyperpolarization of the postsynaptic neurons. There is some evidence of modulation of extrasynaptic GABARs by H^+^, probably via ASIC1 (32). Synaptic GABAARs, including γ^2^ subunits, are benzodiazepine-sensitive, whereas extrasynaptic GABAARs, including δ subunits, which are sensitive to gaboxadol. It is known from pharmaco-electroencephalography studies that the activation of GABAARs by benzodiazepines enhances EEG beta power, and using gaboxadol causes elevated delta power (33). In addition, H^+^ from the extracellular space can activate a specific type of anion channel named PAC (proton-activated outwardly rectifying anion channel), which also mediates the influx of Cl^−^ (34).

**Figure 1:**
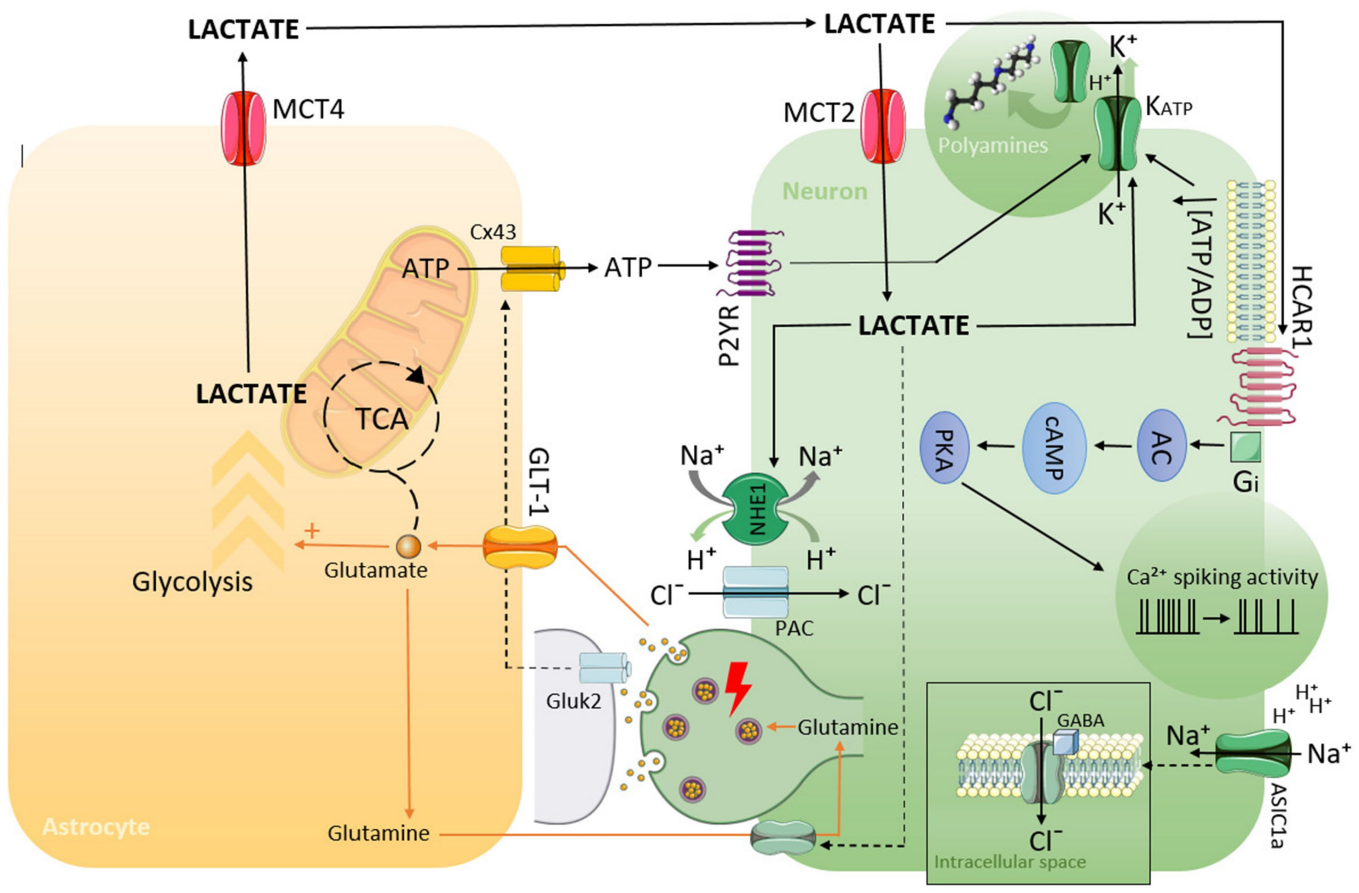
Schematic representation of possible mechanisms by which lactate can lead to hyperpolarization, decreasing neuronal activity, and the generation of EEG slowing as a consequence.

It should be highlighted that the mechanisms described above may act to some extent in each of the discussed disorders (summarized in Figure 1).

For instance, HCA1R is expressed not only in neoplastic tissue and ASIC1a is also involved in the development of different kinds of brain tumors (35).

It could be concluded that the EEG slowing indeed reflects the termination of epileptic discharges. And the mechanisms underlying this phenomenon, at least partially, are lactate-dependent and concentrated in extracellular space. And the study was performed by Skwarzynska et al. (36) that showed the increased extracellular lactate concentration can slow neuronal firing by acting on HCA1R, thereby causing seizure termination, provides a forceful illustration of this point.

## Conclusion

The data summarized in this paper suggest that regional tissue lactacidosis may be a critical factor involved in the pathogenesis of focal slowing in the EEG. The mechanisms by which high extracellular lactate can slow neuronal activity are not simply pH-dependent but suggest its role as a signal molecule. The most important mechanisms involved in the generation of focal slowing are probably the ASIC1a-and HCA1R-lactate signaling pathways. It is also highly possible that the emergence of focal EEG slowing depends on the concentration values of lactate and its persistence in the brain tissue. We believe that focal EEG slowing could be hypothetically considered a “universal” indirect marker of regional brain tissue acidosis.

## Author contributions

DC: Conceptualization, Supervision, Writing – original draft, Writing – review & editing. PP: Writing – review & editing.
